# Evaluation of Clinical Success, Caries Recurrence, and Oral Health-Related Quality of Life of Children Undergoing Full Mouth Rehabilitation for Early Childhood Caries: A Prospective Cohort Study

**DOI:** 10.7759/cureus.50327

**Published:** 2023-12-11

**Authors:** Mebin George Mathew, Ganesh Jeevanandan

**Affiliations:** 1 Pediatric and Preventive Dentistry, Saveetha Dental College and Hospitals, Saveetha Institute of Medical and Technical Sciences, Chennai, IND

**Keywords:** comprehensive dental rehabilitation, primary teeth, full mouth rehabilitation, clinical success, cohort, oral health related quality of life, caries recurrence, early childhood caries, general anesthesia

## Abstract

Aim: This prospective cohort study aimed to assess the clinical success, caries recurrence, and oral health-related quality of life of children undergoing full mouth rehabilitation for early childhood caries under general anesthesia. The study sought to understand the long-term impact of these interventions on early childhood caries (ECC) management.

Methodology: The study included 300 children aged three to six years diagnosed with ECC requiring full mouth rehabilitation under general anesthesia. The assessment included clinical success of various dental treatments, caries recurrence rates, and oral health-related quality of life using the Early Child Oral Health Impact Scale at baseline, 12 months, and 24 months post-treatment. Specialized caries preventive protocols were implemented, including education on oral hygiene, reminders, and nutritional guidance.

Results: 272 children attended the 12-month follow-up, out of which 11 children had new carious lesions. Two hundred fifty-two children were reported for the 24-month follow-up, in which 19 children reported new carious lesions. The clinical success rate of treatment was found to be high. The oral health-related quality of life showed a significant and sustained improvement from baseline to 12 months and further improvement at 24 months post-treatment.

Conclusion: Full mouth rehabilitation under general anesthesia is an effective approach for managing ECC in young children, with high clinical success rates and significant improvements in the oral health-related quality of life over two years. Preventive oral health strategies are necessary to maintain these positive outcomes and improve the overall well-being of affected children.

## Introduction

Early childhood caries (ECC) refers to the presence of a decayed, missing, or filled tooth in children under 71 months of age [1]. Despite being entirely preventable, the prevalence of ECC is rapidly rising, affecting nearly half of preschool-aged children worldwide. ECC stands as the most prevalent chronic disease impacting children today, influencing their growth, development, speech, personality, and academic performance while also constituting a financial burden for their families. It substantially influences the child's and their parents' or caregivers' quality of life [2].

ECC arises from a complex interaction of factors, including diet and feeding practices, tooth structure, oral microbiology, oral hygiene practices, and the child's host defense mechanisms. In its initial stages, ECC is entirely reversible. However, parents often do not seek dental attention for their children at this point, as only a white spot may be visible, and no pain is reported [3]. The enamel of primary teeth is notably thin, making it susceptible to the rapid spread of carious lesions to adjacent teeth if proper oral hygiene practices are not followed, and a diet high in sugars is maintained. The erosion of enamel leads to the formation of definitive cavities, prompting patients to seek dental care due to pain or abscesses. Children may also experience difficulties in eating, and nocturnal pain disrupts their sleep, ultimately affecting their ability to concentrate in school and impeding their academic performance. A visit to the dental office results in missed school days and requires parents to take time off work, leading to financial strain for the family [4,5].

Children afflicted with ECC are often very young and may not readily cooperate with dental procedures. Typically, they seek clinical attention when more invasive treatments like extraction and pulp therapy become necessary. Their limited ability to cooperate can potentially hinder the quality of treatment, making multiple chairside appointments less than ideal for full mouth rehabilitation in cases of ECC [1,4]. To ensure optimal care for children with ECC, full mouth rehabilitation conducted under general anesthesia is considered the preferred approach. This method allows comprehensive treatment to be administered in a condensed timeframe, resulting in high success rates. Consequently, full-mouth rehabilitation under general anesthesia has gained popularity among parents as the preferred means of managing ECC [6].

Undergoing full mouth rehabilitation under general anesthesia leads to a significant enhancement in the oral health-related quality of life (OHRQoL). When appraising the effects of dental general anesthesia, it is crucial to evaluate both the clinical success of the treatment and its impact on OHRQoL. A meta-analysis conducted by Park et al. revealed compelling evidence of short-term improvement in OHRQoL, demonstrating a substantial effect size following full mouth rehabilitation under general anesthesia. It is worth noting that none of the studies included in the analysis had a follow-up period exceeding three months [7].

Additionally, there have been reports of caries recurrence after full mouth rehabilitation under general anesthesia. While some studies have assessed OHRQoL post-rehabilitation, research on treatment's clinical success is limited, and follow-up is minimal [1,3,6-8]. This underscores the critical need to thoroughly understand treatment success and caries recurrence following comprehensive procedures under general anesthesia, especially with a more extended follow-up period. Consequently, this study was undertaken to delve into the impact of full mouth rehabilitation under general anesthesia on ECC.

## Materials and methods

This prospective cohort study commenced after obtaining approval from the Institutional Human Ethics Committee (IHEC/SDC/PhD/PEDO-1620/21/233). The study was conducted from January 2021 to August 2023.

The study included children between the ages of three and six years who were diagnosed with ECC and required full mouth rehabilitation under general anesthesia. Children with special healthcare needs, those outside the specified age range, and those who could be managed in a standard dental office setting were excluded from the study. Out of the 743 children diagnosed with ECC in the outpatient department, 317 patients were initially recruited, out of which 300 willingly participated in the study.

Parents were provided with a thorough explanation of the study and the treatment process by the pediatric dentist and were given ample time to address any queries or concerns. Additionally, all parents had a separate appointment with the anesthesiologist, who detailed the anesthesia delivery procedure and post-operative care. The first appointment encompassed clinical assessments and preoperative interviews. Preoperative radiographs were taken for cooperative patients, whereas uncooperative children had their radiographs captured in the operating room.

The operating team adhered to a standardized treatment protocol for all patients, as outlined in Table 1. Post-operative radiographs for all patients were obtained in the operating room. Both preoperative and postoperative photographs were taken and stored in the dental information archival system of Saveetha Dental College and Hospitals, Saveetha Institute of Medical and Technical Sciences, Chennai.

**Table 1:** Standardized treatment protocol

S. No	Clinical Condition	Proposed Treatment
1	Carious lesions affecting the occlusal surface	Glass ionomer cement (GC Gold Label Type II Universal Restorative, GC Corp., Tokyo, Japan) OR composite (Tetric N-Ceram, Ivoclar Vivadent, Schaan, Liechtenstein).
2	Primary molar teeth with multisurface carious lesions	Stainless Steel Crown (3M™ ESPE™, Saint Paul, MN, United States)
			3	Anterior teeth with multi-surface lesions	Strip crowns (3M™ ESPE™, Saint Paul, MN, United States)
4	Teeth with pulpal involvement	American Academy of Pediatric Dentistry guidelines, followed by anterior teeth were restored with strip crowns (3M™ ESPE™, Saint Paul, MN, United States) and posterior teeth with stainless steel crowns (3M™ ESPE™, Saint Paul, MN, United States)			
5	Teeth with necrotic pulps and non-restorable teeth	Extraction. Space maintainers were placed based on clinical and radiographic findings

Data collection for OHRQoL

In this study, the assessment of OHRQoL was conducted using the Early Child Oral Health Impact Scale (ECOHIS), a validated and reliable scale for preschool-aged children [9]. ECOHIS comprises two sections: the Child Impact Section (CIS) and the Family Impact Section (FIS), consisting of 13 questions parents answer. Scores on this scale range from zero to 52, where higher scores indicate a higher number of problems and, consequently, lower OHRQoL. OHRQoL was evaluated at baseline, 12 months, and 24 months.

Clinical success of dental treatment

**Figure 1:**
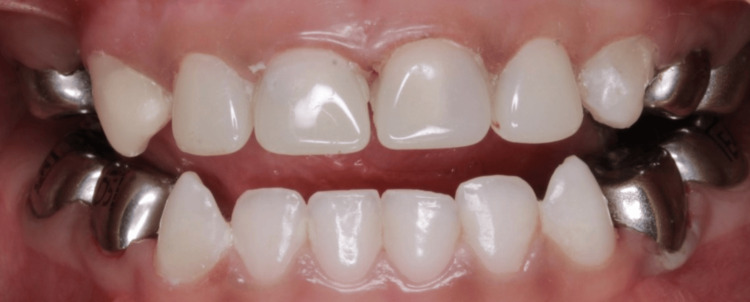
Successful treatment at follow up visit.

After both 12 and 24 months post-treatment, parents received a reminder via phone call for the follow-up visit to evaluate clinical success and OHRQoL. The clinical examination was conducted by the same pediatric dentist who had initially performed the baseline assessment in the dental office (Figure 1). If radiographs were deemed necessary during this visit, they were taken provided the child was cooperative. The examining pediatric dentist was unaware of which had carried out the full mouth rehabilitation under general anesthesia.

Much like the baseline examination, the examiner remained unaware of the specific provider who administered the treatment in the operating room. The criteria for clinical failure [6,8], as applied in this study, have been outlined in Table 2.

**Table 2:** Clinical Success Criteria

Restorative Procedures
The restoration or procedure was considered a failure if one or more of the following was present:
• Recurrent caries around restorations.
• Missing, fractured, cracked or poorly adapted restorations.
• Open margins, perforated or missing SSC/strip crowns/ zirconia crowns.
• Restored tooth extracted/lost due to pathology.
• Dislodgement
•Tooth extracted due to pathology
•Tooth mobility
Pulp Therapy
Pulp therapy was considered failure if the tooth has one of the following symptoms:
• Sensitivity to percussion.
• Localized pain.
• Presence of swelling or abscess.
• Radiographic evidence of interradicular pathology
•Tooth extracted due to pathology
•Tooth mobility
Space maintainer
Space maintainer was considered failure if one or more of the following was present
• missing space maintainer.
•fractured space maintainer

Caries recurrence after full mouth rehabilitation

**Figure 2:**
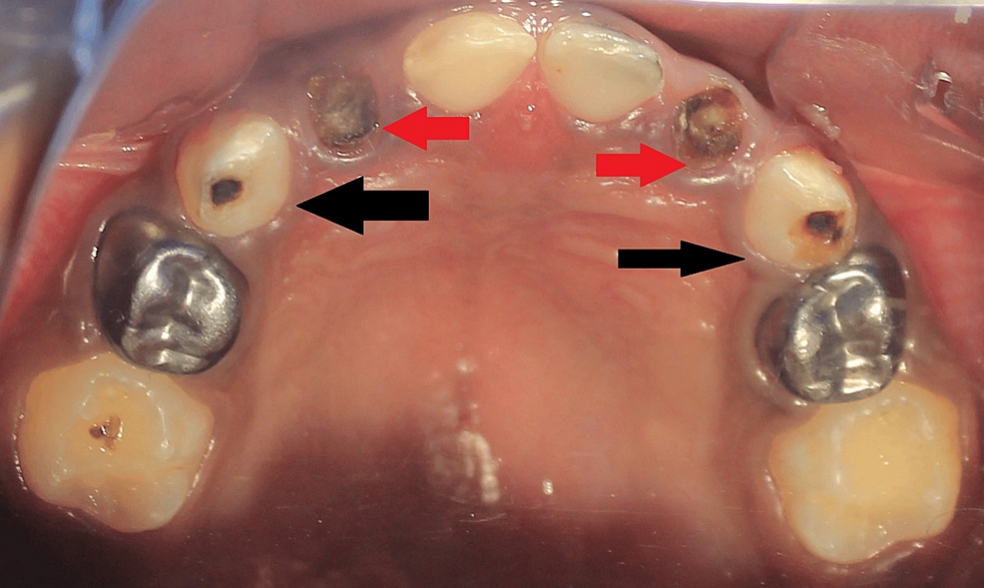
Failed treatment at follow-up visit The red arrow depicts dislodged strip crowns in primary lateral incisors. The black arrow depicts a new carious lesion in primary canines.

The emergence of a new carious lesion on a tooth that showed no signs of decay during the initial visit was categorized as a recurrence of caries (Figure 2). If a new carious lesion appeared on the border of an existing restoration, it was designated secondary caries. The occurrence of caries recurrences ultimately resulted in an increase in the decayed, missing, and filled teeth index compared to the baseline assessment.

The collected data underwent statistical analysis using the Statistical Package for the Social Sciences version 28 (SPSS, Inc., Chicago, Ill., USA). A significance level of P < 0.05 was deemed statistically significant. The Chi-square test and Fisher’s exact test were employed to compare the success rates of dental treatments before and after the intervention.

## Results

The present prospective cohort study involved 300 children who required full mouth rehabilitation under general anesthesia. Follow-up assessments were conducted at 12 and 24 months. The study cohort comprised 148 boys and 152 girls, with an average age of 4.2±0.7. Among the parents, 158 had attained a post-secondary or higher educational qualification. Additionally, 161 children came from families with an annual income exceeding ₹300,000 (Table 3).

**Table 3:** Parent and child characteristics at baseline dmft: number of decayed, missing and filled primary teeth

Parent and child characteristics	Frequency	Percentage
PARENT DEMOGRAPHICS		
Relationship to the child		
Mother	201	67%
Father	99	33%
Education level		
Secondary or below	142	49.33%
Post-Secondary or above	158	52.67%
Family Income (₹ per year)		
<300,000	139	46.33%
>300,000	161	53.67%
CHILD DEMOGRAPHICS		
Gender		
Male	148	49.33%
Female	152	50.67%
Age (years), Mean (± SD)	4.2(0.7)	-
Child’s dental status		
dmft score, mean (± SD)	11.1(1.9)	-

**Figure 3:**
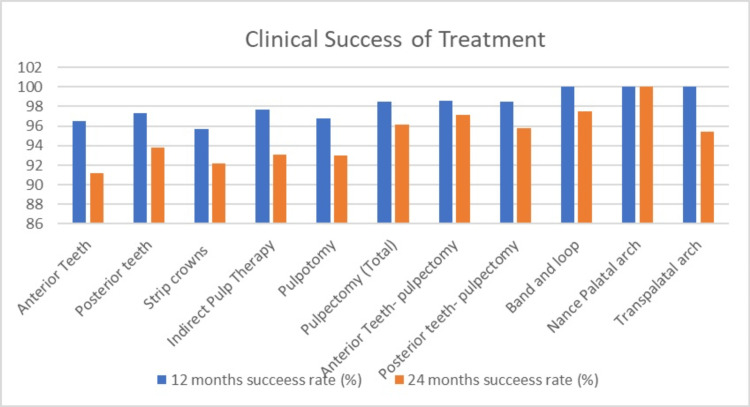
Clinical success rate of treatment

In this study, the Nance palatal arch demonstrated the highest success rate, achieving 100% effectiveness at 12- and 24-month follow-ups. Stainless steel crowns closely followed, with a success rate of 99.81% at 12 months and 99.62% at 24 months (Table 4, Figure 3). Notably, pulpectomy emerged as the most frequently performed treatment.

**Table 4:** The success rates of the treatment procedures at the 12 and 24-month follow-up after full mouth rehabilitation under general anesthesia %: percentage

Treatment	Total Number of Teeth	12 months		24 months	
		No of successful treatment	Success rate (%)	No of successful treatment	Success rate (%)
Restorative procedures					
Anterior Teeth	113	109	96.46	103	91.15
Posterior teeth	226	220	97.34	212	93.80
Crowns					
Strip crowns	652	624	95.70	601	92.17
SSC	1579	1576	99.81	1573	99.62
Pulp Therapy					
Indirect Pulp Therapy	87	85	97.70	81	93.10
Pulpotomy	186	180	96.77	173	93.01
Pulpectomy (Total)	1613	1589	98.51	1551	96.15
Anterior Teeth- pulpectomy	427	421	98.59	415	97.18
Posterior teeth- pulpectomy	1186	1168	98.48	1136	95.78
Space Maintainer					
Band and loop	79	79	100	77	97.46
Nance Palatal arch	27	27	100	27	100
Transpalatal arch	22	22	100	21	95.45

Among the 272 children who attended the 12-month follow-up, 11 reported a recurrence of caries. At the 24-month follow-up, 19 out of 252 children exhibited new carious lesions. A statistically significant gender-based difference (P < 0.05) in caries recurrence was observed at 24 months. Furthermore, a statistically significant difference (P < 0.05) in caries recurrence was noted between families with an income less than ₹300,000 and those with an income greater than ₹300,000 (Table 5).

**Table 5:** Caries recurrence rate at 12-month and 24-month follow up SD: standard deviation; N: number; %: percentage; dmft: number of decayed, missing and filled primary teeth; dmfs: number of surfaces of decayed, missing and filled primary teeth; A significance level of P < 0.05 was deemed statistically significant

Variable	12 months follow up			24 months follow up		
	(n=283)			(n=271)		
	No relapse	Relapse	P value	No relapse	Relapse	P value
	(n=272)	(n=11)		(n=252)	(n=19)	
Age at treatment (year, mean (SD))	4.4±0.8	4.1±0.6	0.8	4.4±0.9	4.2±0.8	0.8
Sex (N (%))						
Male	138 (50.7)	6 (54.5)	0.7	129 (51.1)	13(68.4)	<0.05
Female	134 (49.3)	5 (45.5)		123 (48.9)	6 (31.6)	
Family annual income(₹) (N (%))						
<300,000	148 (54.4)	6 (54.5)	0.9	134 (54.2)	14(73.7)	<0.05
>300,000	124 (45.6)	5 (45.5)		118 (46.8)	5 (26.3)	
Area of residence (N (%))						
Urban	211 (77.6)	8 (72.7)	0.9	198 (78.6)	14 (73.7)	0.8
Rural	61 (22.4)	3 (27.3)		54 (21.4)	5(26.3)	
Baseline dmft score (mean (SD))	9.1 (3.9)	12.1(4.8)	<0.05	9.9 (4.2)	14.9(3.5)	<0.001
Baseline dmfs score (mean (SD))	28.6 (16.2)	36.5 (18.1)	<0.001	31.4 (19.1)	48.8 (16.7)	<0.001

**Figure 4:**
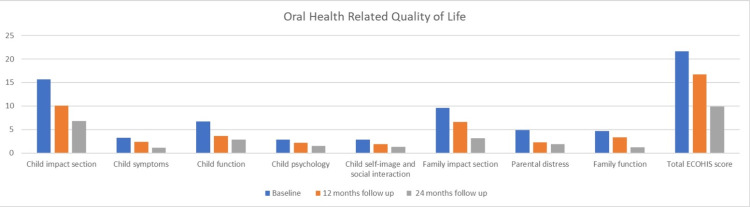
Mean Scores of Early Childhood Oral Health Impact Scale representing Oral Health-Related Quality of Life ECOHIS: Early Childhood Oral Health Impact Scale

The evaluation of OHRQoL was conducted using the ECOHIS scale. The score in the Child Impact section decreased from 15.7±4.1 at baseline to 6.8±1.9 at the end of 24 months. Similarly, the Family Impact section score reduced from 9.6±2.7 to 3.1±2.6. These reductions in scores were highly statistically significant (P < 0.001*). The overall ECOHIS score also showed a notable decrease, dropping from 21.6±9.5 at baseline to 16.7±4.1 at 12 months, and further reducing to 9.9±4.2 by the end of 24 months. This difference was highly statistically significant (P < 0.001*). It was observed that treatment under general anesthesia significantly improved OHRQoL ( Figure 4, Table 6).

**Table 6:** Mean scores and mean differences in scores the early childhood oral health impact scale before and after dental rehabilitation under general anesthesia SD: standard deviation; ECOHIS: Early childhood oral health impact scale; A significance level of P < 0.05 was deemed statistically significant

ECOHIS domains	No. of items	Baseline (±SD)	At 12 months follow up (±SD)	At 24 months follow up (±SD)	P-value
Child impact section	9	15.7±4.1	10.1±1.6	6.8±1.9	< .001
Child symptoms	1	3.2±0.9	2.4±0.3	1.1±0.6	< .001
Child function	4	6.7±3.2	3.6±1.1	2.9±1.2	< .001
Child psychology	2	2.9±1.7	2.2±0.1	1.5±1.2	< .001
Child self-image and social interaction	2	2.9 ±1.8	1.9±0.2	1.3±1.2	< .001
Family impact section	4	9.6±2.7	6.6±2.5	3.1±2.6	< .001
Parental distress	2	4.9±2.3	2.3±1.1	1.9±1.2	< .001
Family function	2	4.7±1.1	3.3±1.4	1.2±0.8	< .001
Total ECOHIS score	13	21.6±9.5	16.7±4.1	9.9 ±4.2	< .001

## Discussion

In this study, we assessed the clinical success of treatment, caries recurrence, and OHRQoL. The majority of treatments showed notable success, leading to a significant improvement in OHRQoL. However, there were instances of caries recurrence in a few children at one-year and two-year follow-up visits. Our findings contrast with those of Graves et al. [10], who reported that managing children under general anesthesia did not always yield acceptable outcomes. Conversely, Amin et al. [11] reported early positive outcomes after full mouth rehabilitation under general anesthesia.

Specifically, the clinical success rates for anterior restorations were 96.46% at one year and 91.05% for anterior composites after two years. For posterior teeth, the rates were 97.34% at 12 months and 93.80% at 24 months. This differs from the rates reported by Lin and Lin, who found 71.7% and 90.3% success rates for anterior and posterior composites, respectively [12]. Eshghi et al. reported success rates of 86.2% and 93.4% for anterior and posterior composites at the end of 24 months [13]. One possible reason for the higher success rate in our study could be attributed to advancements in restorative materials over the past decade. Additionally, the higher success rate for posterior teeth might be attributed to the fact that anterior teeth are subjected to more forces and are prone to fracture during food-cutting activities in children [6]. Khodadadi et al. reported a failure rate of 9.44% for anterior teeth, which aligns with the findings of our current study [14].

The integrity of primary maxillary anterior teeth plays a pivotal role in both appearance and function. Their damage or loss raises aesthetic concerns and can lead to parafunctional habits such as tongue thrusting, speech difficulties, psychological stress, and diminished chewing effectiveness. Resin composite strip crowns are employed to restore primary incisors affected by extensive or multi-surface caries, provided there is adequate remaining tooth structure following the removal of all decayed tissue [8].

In the current study, strip crowns demonstrated a success rate of 95.70% at 12 months and 92.17% at two years. This differs from the findings of Tate et al. (51.9%) [15] and Lin and Lin (71.7%) [12] but aligns with the results of Zhao et al., with 95.92% at one year and 93.42% at two years [6]. The success rate of anterior composite restorations in our study closely mirrored that of strip crowns. This could be attributed to the fact that the operator selected either an anterior composite or strip crown based on the amount of remaining tooth structure. Since these technique-sensitive strip crowns were placed under general anesthesia, achieving proper isolation was more straightforward, potentially contributing to the higher success rates.

Stainless steel crowns have been used for over 75 years in rehabilitating primary molars with multi-surface carious lesions and after pulp therapy [16]. In our study, the success rate for stainless steel crowns was notably high, standing at 99.81% at 12 months and 99.62% at 24 months. This success rate slightly surpasses that reported by Khodadi (94.74%) [14], Jiang (95.1%) [8], Schuler (97.2%) [17], and Zhao (97.26%) [6]. The remarkable success of stainless steel crowns can be attributed to their protective function. Teeth with restorations are potential areas for new carious lesions, which are less likely to occur when the tooth is fitted with a crown [16]. The occasional failure of stainless steel crowns may be attributed to inadequate pulpal treatment or resorption of the primary teeth.

In the present study, pulp therapy demonstrated a notably high success rate, aligning with findings from previously published studies. The success rates for pulpectomy at 12 and 24 months were 98.51% and 96.15%, respectively. Interestingly, the success rates for anterior teeth at 12 and 24 months were slightly higher than posterior teeth. This discrepancy can be attributed to the intricate root canal system of primary molars in contrast to the single-rooted structure of anterior teeth [18]. The presence of accessory canals in primary molars may lead to more challenges in inflammation control, potentially resulting in a higher incidence of failures. Additionally, the "hollow tube effect" associated with the use of Metapex as the obturating material could contribute to pulpectomy failures in primary teeth, as it provides a conducive environment for microorganisms to thrive within unfilled roots [6].

Children undergoing full-mouth rehabilitation under general anesthesia are deemed to be at a heightened risk for dental caries. It is important to note that treatment under general anesthesia is not the primary approach for ECC. Instead, a comprehensive strategy emphasizing preventive modifications in dietary habits, oral hygiene practices, and regular post-treatment dental check-ups is typically preferred [19,20]. However, reports of caries relapse after dental treatment under general anesthesia are not uncommon. This can be attributed to inadequate adherence to post-operative diet and oral hygiene instructions by both the parent and child and a failure to attend follow-up appointments [21]. ECC has a complex etiology, and the development of new carious lesions may occur due to microbial adhesion to newly placed prosthetics like stainless steel crowns and space maintainers, particularly if proper oral hygiene is not maintained [19].

Despite implementing preventive measures during the procedure and maintaining strict post-operative precautions, caries recurrence was observed in our study. Specifically, the recurrence rates were 3.88% at 12 months and 7.01% at 24 months. Our results showed lower caries recurrence compared to the rates reported by Amin et al. (29%) [11], Jiang et al. (18.8%) [8], and Zhao et al. (37.23%) [6] at the 12-month mark. Additionally, the recurrence rate at 24 months was much lower in comparison to the results reported by Foster et al. (53%) [22], El-Batawi (58.5%) [23], and Almeida (79%) [24]. The variation in recurrence rates can be attributed to the complex etiology of ECC and differences in the treatment plans and preventive protocols followed by each research team [1,23]. 

Our study's low caries recurrence rates can be credited to the specialized caries preventive protocol established to minimize the risk of caries relapse after full mouth rehabilitation. This protocol included education on oral hygiene, regular reminders, and nutritional guidance to ensure that both the child and their parents could maintain good oral hygiene practices, thereby preventing the development of new carious lesions. The high success rate of the administered treatment may also contribute to the low rate of caries recurrence.

One of the contributing factors to the growing popularity of full-mouth rehabilitation is the notable enhancement of OHRQoL following the procedure [1,7,19]. Our present study observed a significant improvement at both the 12 and 24-month marks. Notably, the OHRQoL displayed a substantial upswing from baseline to 12 months, and then from 12 months to 24 months. This positive change can be attributed to several factors. This contrasts with the findings of Jiang et al. [8], who noted a significant improvement in OHRQoL post-treatment but observed a decline after three months. In our study, the significant and sustained improvement may be attributed to the high treatment success rate and the low recurrence of caries. Additionally, implementing our specialized caries control protocol, involving weekly discussions between parents and the dental team regarding oral health and hygiene practices, likely played a pivotal role. This underscores the potential of full mouth rehabilitation under general anesthesia to lead to long-term enhancements in OHRQoL and overall oral health, provided that preventive measures are diligently followed.

The present study has its own set of limitations. The cohort was comprised of 300 children treated at a single university hospital, which means that the findings may not be applicable on a broader scale. Additionally, a small number of children did not attend their follow-up appointments at 12 and 24 months, potentially impacting the assessment of treatment success and caries recurrence rate. However, the study also exhibits notable strengths. These include its prospective cohort design, high retention rate, significant clinical success rate, and low caries recurrence rate. These results can prove beneficial for those involved in formulating oral health policies for children. They offer valuable insights for prioritizing patients' treatment and ensuring regular follow-up visits, including routine preventive dental therapy.

## Conclusions

This prospective cohort study highlights the importance of addressing ECC in young children through full-mouth rehabilitation under general anesthesia. The findings reveal a high clinical success rate for various dental treatments, including stainless steel crowns and pulpectomy, suggesting that this approach can effectively manage ECC. The study also demonstrates a significant improvement in the OHRQoL for children over two years after the procedure. This not only underscores the positive impact of full mouth rehabilitation on the well-being of affected children but also highlights the potential long-term benefits if effective preventive measures are maintained.

Furthermore, the study emphasizes the need for ongoing efforts in preventive oral health strategies. The relatively low caries recurrence rates in this study suggest that a specialized caries control protocol, regular follow-up appointments, and patient education can contribute to sustaining improved OHRQoL.
